# Autologous peripheral blood stem cell mobilization following dose-adjusted cyclophosphamide, doxorubicin, vincristine, and prednisolone chemotherapy alone or in combination with rituximab in treating high-risk non-Hodgkin’s lymphoma

**DOI:** 10.1186/s40880-015-0045-3

**Published:** 2015-09-14

**Authors:** Yuankai Shi, Ping Zhou, Xiaohong Han, Xiaohui He, Shengyu Zhou, Peng Liu, Jianliang Yang, Changgong Zhang, Lin Gui, Yan Qin, Sheng Yang, Liya Zhao, Jiarui Yao, Shuxiang Zhang

**Affiliations:** Department of Medical Oncology, Cancer Hospital, Chinese Academy of Medical Sciences & Peking Union Medical College, Beijing Key Laboratory of Clinical Study on Anticancer Molecular Targeted Drugs, Beijing, 100021 P. R. China

**Keywords:** Stem cell transplantation, Mobilization, CHOP regimen, Rituximab, Non-Hodgkin’s lymphoma

## Abstract

**Background:**

The regimen of cyclophosphamide, doxorubicin, vincristine, and prednisolone (CHOP) is an efficient treatment of non-Hodgkin’s lymphoma (NHL). This study aimed to assess the efficacy and toxicity of dose-adjusted CHOP alone or in combination with rituximab (R-CHOP) by examining the stem cell mobilization in NHL patients. Factors affecting the collection of CD34^+^ cells were also explored.

**Methods:**

Our retrospective study included 39 patients eligible for autologous stem cell transplantation: 14 patients who expressed CD20 and were financially eligible received R-CHOP for autologous peripheral blood stem cell (APBSC) mobilization; the remaining 25 patients received CHOP.

**Results:**

The median CD34^+^ cell yield was 7.01 × 10^6^ cells/kg body weight (range 1.49–28.39 × 10^6^ cells/kg body weight), with only two patients failing to meet the target CD34^+^ cell harvest of ≥2.0 × 10^6^ cells/kg body weight. The median number of apheresis procedures per patient was 1 (range 1–3). The APBSC mobilization yield of the CHOP group appeared to be higher than that of the R-CHOP group (*P* = 0.005), whereas the success rate was similar between groups. R-CHOP elevated the complete response (CR) rate in B cell lymphoma patients as compared with CHOP (*P* = 0.01). No significant differences in toxicity or engraftment were observed between the two groups.

**Conclusion:**

The present study demonstrated that dose-adjusted CHOP chemotherapy effectively mobilized APBSCs in NHL patients and that the addition of rituximab to dose-adjusted CHOP chemotherapy elevated the CR rate for patients with B-cell lymphoma.

## Background

Autologous stem cell transplantation (ASCT) provides hematopoietic support after high-dose chemotherapy and is widely used to treat non-Hodgkin’s lymphoma (NHL). Successful stem cell collection is a prerequisite for ASCT. The combination of chemotherapy with recombinant human granulocyte colony-stimulating factor (rhG-CSF) is a commonly used mobilization strategy, resulting in higher CD34^+^ cell yields than mobilization with rhG-CSF alone [1, 2]. This approach also purges the mobilized tumor cells, reducing the tumor burden, and leads to chemosensitivity before transplantation. Nevertheless, additional study is required to determine the optimal mobilization regimen in terms of safety, progenitor yield, engraftment, and contamination with tumor cells [3].

Various chemomobilization regimens have been widely used in patients with lymphoma. High-dose cyclophosphamide, high-dose etoposide, and platinum-based chemotherapies such as ifosfamide, carboplatin, etoposide (ICE); cisplatin, cytarabine, dexamethasone (DHAP); and etoposide, methylprednisolone, cytarabine, cisplatin (ESHAP) are commonly used in combination with rhG-CSF [4]. Nevertheless, all current regimens fail to mobilize a sufficient number of hematopoietic stem cells to proceed to transplantation in 5–40% of patients [1, 2, 5], and some patients relapse after ASCT due to residual disease after high-dose chemotherapy or contamination of the mobilization product with tumor cells [6, 7]. Therefore, additional research is required to determine a more effective mobilization regimen to improve patients’ outcome and survival.

Rituximab is commonly used in the first- or second-line therapy of NHL patients before autologous peripheral blood stem cell (APBSC) mobilization as a way to improve the in vivo purging of circulating lymphoma cells before stem cell collection [8]. However, whether rituximab compromises the yield of APBSCs and the hematopoietic recovery after ASCT remains a topic of debate [6, 8–15]. The regimen of cyclophosphamide, doxorubicin, vincristine, and prednisolone (CHOP) has been established as the standard treatment for NHL [16]. However, few studies have explored APBSC mobilization following CHOP combined with rituximab (R-CHOP) in high-risk NHL. Therefore, the present retrospective study was designed to analyze the efficacy and toxicity of dose-adjusted CHOP or CHOP-like regimen alone or in combination with rituximab for APBSC mobilization and to explore the factors that may affect the yield of CD34^+^ cells.

## Patients and methods

### Patient selection

Patients diagnosed with stage III/IV, highly aggressive B-cell lymphoma or T-cell lymphoma between August 2000 and June 2013 were identified from a database of prospective observational research on ASCT in malignant lymphoma. The analysis was approved by the Institutional Review Board of the Cancer Institute/Hospital, Chinese Academy of Medical Science and Peking Union Medical College (CAMS and PUMC). All patient diagnoses were histologically confirmed at the Department of Pathology, Cancer Institute/Hospital, CAMS and PUMC. All patients were required to be younger than 65 years old and to have adequate organ function. Patients were excluded if they had human immunodeficiency virus (HIV) infection, central nervous system (CNS) disease, active hepatitis B or C, or grade ≥2 peripheral neuropathy. Patients were staged according to the Ann Arbor Classification before mobilization. Staging procedures included physical examination, reporting of B symptoms, computer tomography (CT) or positron emission tomography (PET)-CT scans, and bone marrow biopsy. Because this was a retrospective study, the use of PET-CT to aid in the assessment of the response had varied over time. All patients exhibited at least a partial response (PR) at the time of mobilization and were on their first mobilization attempt.

### APBSC mobilization

Patients were treated with induction chemotherapy regimens such as CHOP; bleomycin, epirubicin, cyclophosphamide, vincristine, plus prednisone (BACOP); and prednisone, doxorubicin, cyclophosphamide, etoposide, cytarabine, bleomycin, vincristine, plus methotrexate (ProMACE/CytaBOM) before mobilization.

Mobilization chemotherapy included dose-adjusted CHOP or CHOP-like regimens alone (CHOP group) or in combination with rituximab (R-CHOP group) according to CD20 expression and patients’ financial circumstances. The dose-adjusted CHOP regimen consists of cyclophosphamide (1–2 g/m^2^ per day), vincristine (1.4 mg/m^2^ per day, maximum 2 mg), and doxorubicin (50 mg/m^2^ per day) or epirubicin (75 mg/m^2^ per day) all on day 1 and prednisone (100 mg/day) on days 1–5. The CHOPE regimen consists of CHOP plus etoposide (100–200 mg/m^2^ per day) on days 1–3. The BACEP regimen consists of bleomycin (15 mg/day) on days 3 and 10, epirubicin (75 mg/m^2^ per day) on day 1, cyclophosphamide (1 g/m^2^ per day) on day 1, etoposide (100 mg/m^2^ per day) on days 1–3, prednisone (100 mg/day) on days 1–5. Rituximab was administered at 375 mg/m^2^ per day on the day before mobilization, day 7 after mobilization, the day before APBSC infusion, and day 8 after infusion [17].

The first dose of rhG-CSF was administered subcutaneously at a fixed dose of 300 µg/day (150 µg/day for patients weighing less than 45 kg) beginning on the day when the white blood cell (WBC) count first rose after the nadir following chemotherapy, continuing until the day before the last apheresis. To determine the first day of apheresis, the WBC count and the percentage of CD34^+^ cells in peripheral blood (defined as the ratio of CD34^+^ cells to mononuclear cells [MNCs]) were monitored daily after chemotherapy. When the WBC count exceeded 10 × 10^9^/L, the MNC count exceeded 2 × 10^9^/L, and the percentage of CD34^+^ cells in peripheral blood exceeded 1% [18], continuous APBSC collection was conducted daily with a CS-3000 Plus blood cell separator (Baxter Healthcare Corp., Deerfield, IL, USA) until a target collection of at least 2 × 10^6^ CD34^+^ cells/kg body weight or 4 × 10^8^ MNCs/kg body weight was achieved. The blood volume processed by each single apheresis was 110–150 mL/kg body weight at a speed of 40–70 mL/min. Venous access was obtained by a double lumen catheter (Arrow International Inc., Reading, PA, USA) placed in a femoral vein [17, 18]. After mobilization, the amount of CD34^+^ cells in the apheresis product was measured after each collection by flow cytometry using a class III monoclonal antibody (Becton–Dickinson, Franklin Lakes, NJ, USA) [19]. Dimethylsulfoxide was added to the products at a final concentration of 10% to protect the cells from the stress or death caused by cryopreservation. The products were stored at −80°C with an uncontrolled freezing rate. Twenty-four hours later, the samples were transferred into a liquid nitrogen container (Thermo Scientific, Waltham, MA, USA) and stored at −196°C [18].

Successful mobilization was defined as the collection of a minimum of 2 × 10^6^ CD34^+^ cells/kg body weight in a single mobilization. Optimal mobilization was defined as 5 × 10^6^ CD34^+^ cells/kg body weight collection in a single mobilization [20]. Failed mobilization was defined as the failure to collect at least 2 × 10^6^ CD34^+^ cells/kg body weight by aphaeresis.

### Conditioning regimen and engraftment

The conditioning regimens were BEAC [carmustine (300 mg/m^2^, day −5), etoposide (800 mg/m^2^, days −4 to −2), cytarabine (1600 mg/m^2^, days −4 to −2), cyclophosphamide (1.8 g/m^2^, days −6 to −5)], BEAM [carmustine (300 mg/m^2^, day −5), etoposide (800 mg/m^2^, days −4 to −2), cytarabine (1600 mg/m^2^, days −4 to −2), melphalan (140–160 mg/m^2^ for oral administration, day −6)], CBV [cyclophosphamide (1.8 g/m^2^, days −3 to −2), carmustine (450–600 mg/m^2^, day −7), etoposide (900–1600 mg/m^2^, days −6 to −4)], and CE-TBI [cyclophosphamide (1.8 g/m^2^, days −3 to −2), etoposide (750 mg/m^2^, days −6 to −4), total body irradiation (800–900 Gy, day −7)]. The day APBSCs were infused was defined as day 0. rhG-CSF (300 µg/day) was administered after APBSC infusion on day 6 and was continued until the neutrophil count recovered to at least 0.5 × 10^9^/L on two consecutive days, defined as neutrophil engraftment. Platelet (PLT) engraftment was taken as the time when the PLT count recovered to more than 50 × 10^9^/L for two consecutive days without transfusion support.

### Evaluation of mobilization responses, toxicities, and survival

Response to treatment was evaluated according to the International Workshop Criteria [21], and all adverse reactions were recorded and graded according to the National Cancer Institute criteria [22]. Overall survival (OS) and progression-free survival (PFS) were measured from the date of mobilization to the end of follow-up. Routine follow-up by imaging analysis was performed every 3 months for the first 2 years, every 6 months for the next 3 years, and then annually or whenever clinically indicated. The last follow-up was on November 30, 2013.

### Statistical methods

All calculations and statistical analyses were conducted using SPSS software (version 19.0, SPSS Inc., Chicago, IL, USA). For quantitative variables, medians, ranges, and proportions were determined and analyzed by descriptive statistics and frequency analysis. Comparisons of categorical variables between groups were tested by the Chi square test or Fisher’s exact test, and continuous variables were compared between two groups by the Mann–Whitney *U* test. For univariate analysis, a Spearman correlation analysis was conducted for continuous variables, and a Mann–Whitney *U* test was conducted for categorical variables to explore the effect of pre-mobilization factors on the yield of CD34^+^ cells. A linear stepwise regression was used for multivariate analysis. Survival data were analyzed using the Kaplan–Meier method, and survival curves were compared using the log-rank test. A two-tailed *P* value of <0.05 was considered significant.

## Results

### Patients

A total of 39 patients were included in the analysis, with a median age of 33 (range 11–57) years. CHOP or CHOP-like regimens were administered for APBSC mobilization. Of the 25 patients in the CHOP group, 15 received CHOP regimen, 8 received CHOPE regimen, and 2 received BACEP regimen; of the 14 patients in the R-CHOP group, 11 received R-CHOP regimen, and 3 received R-CHOPE regimen. The median number of chemotherapy cycles prior to mobilization was 4 (range 2–6). As a result, 17 patients (43.6%) achieved a CR, and 22 (56.4%) achieved a PR prior to mobilization. The R-CHOP and CHOP groups were well matched in terms of age, sex, Ann Arborstage, age-adjusted international prognostic index (aaIPI), previous chemotherapy cycles, and disease status at mobilization (Table 1). All T-cell lymphoma patients were included in the CHOP group, though tumor origin did not affect the CD34^+^ cell mobilization yield (*P* = 0.061).

**Table 1:** Baseline characteristics of 39 patients with high-risk non-Hodgkin’s lymphoma

Characteristic	R-CHOP [cases (%)]	CHOP [cases (%)]	*P* value
Total	14	25	
Sex			0.673
Male	8 (57.1)	16 (64.0)	
Female	6 (42.9)	9 (36.0)	
Tumor origin			<0.001
B-cell	14 (100.0)	8 (32.0)	
T-cell	0	15 (60.0)	
NA	0	2 (8.0)	
Ann arbor stage			0.119
I/II	1 (7.1)	8 (32.0)	
III/IV	13 (92.9)	17 (68.0)	
ECOG score			0.289
<2	13 (92.9)	23 (92.0)	
≥2	1 (7.1)	2 (8.0)	
LDH level (IU/L)			0.328
>225	9 (64.3)	12 (48.0)	
≤225	5 (35.7)	13 (52.0)	
B symptoms			0.089
No	5 (35.7)	16 (64.0)	
Yes	9 (64.3)	9 (36.0)	
Bulky disease			0.431
No	6 (42.9)	14 (56.0)	
Yes	8 (57.1)	11 (44.0)	
Extranodal invasion			0.652
No	3 (21.4)	7 (28.0)	
Yes	11 (78.6)	18 (72.0)	
aaIPI			0.096
0–1	4 (28.6)	15 (60.0)	
2–3	10 (71.4)	10 (20.0)	
Disease status before mobilization			0.201
CR	8 (57.1)	9 (36.0)	
PR	6 (42.9)	16 (64.0)	
Previous radiation therapy	0	2 (8.0)	0.528

*R-CHOP* rituximab combined with cyclophosphamide, doxorubicin, vincristine, and prednisolone (CHOP) or CHOP-like regimen; CHOP, CHOP or CHOP-like regimen without rituximab; *ECOG* Eastern Cooperative Oncology Group; *LDH* lactate dehydrogenase; *aaIPI* age-adjusted International Prognostic Index; *CR* complete response; *PR* partial response; *NA* not available

### APBSC mobilization

For the 39 patients, the median CD34^+^ cell yield was 7.01 × 10^6^ cells/kg body weight, with only 2 patients failing to meet the target CD34^+^ cell harvest of ≥2.0 × 10^6^ cells/kg body weight (1.46 × 10^6^ and 1.61 × 10^6^ cells/kg body weight, respectively), both of whom underwent successful ASCT. The optimal mobilization was achieved in 27 patients (69.2%), and 19 of them only underwent one apheresis procedure with a median CD34^+^ cell collection of 7.48 × 10^6^ cells/kg body weight (range 5.03–18.50 × 10^6^ cells/kg body weight).

The time from mobilization to apheresis was longer in the R-CHOP group than in the CHOP group (*P* = 0.032), whereas the median number of apheresis procedures was similar between the two groups. The percentage of CD34^+^ cells in peripheral blood by the first apheresis, the WBC count, and the mobilization yield of CD34^+^ cells were significantly lower in the R-CHOP group than in the CHOP group. Excluding the confounding factor tumor origin, the mobilization yield of CD34^+^ cells for B-cell lymphoma patients was still significantly higher in the CHOP group than in the R-CHOP group (*P* = 0.029). However, the rate of optimal mobilization between the two groups was similar (*P* = 0.075). More details are outlined in Table 2.

**Table 2:** Outcomes and efficacy of mobilization in 39 patients with high-risk non-Hodgkin’s lymphoma

Parameter	All patients (*n* = 39)	R-CHOP (*n* = 14)	CHOP (*n* = 25)	*P* value
Time from mobilization chemotherapy to rhG-CSF support (days)^a^	9 (6–15)	12.5 (9–15)	12 (6–15)	0.082
Duration of rhG-CSF support (days)^a^	5 (3–10)	5 (3–9)	5 (4–10)	0.343
Time from mobilization chemotherapy to apheresis (days)^a^	16 (12–20)	16.5 (15–18)	16 (12–20)	0.032
Percentage of CD34^+^ cells in peripheral blood on the first day of apheresis (%)^a^	2.43 (0.36–7.50)	1.35 (0.36–3.41)	3.14 (1.14–7.50)	0.002
Peripheral blood WBC count on the first day of apheresis (×10^9^/L)^a^	14.30 (3.55–43.20)	10.35 (3.55–43.20)	17.15 (5.56–41.60)	0.031
Number of apheresis procedures^a^	1 (1–3)	1 (1–2)	1 (1–3)	0.593
CD34^+^ cells collected on day 1 (×10^6^ cells/kg body weight)^a^	5.79 (0.40–18.50)	4.83 (1.03–8.11)	7.07 (0.40–18.50)	0.016
CD34^+^ cells collected in total (×10^6^ cells/kg body weight)^a^	7.01 (1.49–28.39)	5.01 (1.49–13.40)	8.25 (1.61–28.39)	0.005
CD34^+^ cells collected per apheresis (×10^6^ cells/kg body weight)^a^	5.97 (0.75–18.50)	4.83 (0.75–8.11)	7.31 (0.81–18.50)	0.014
Optimal mobilization of CD34^+^ cell collection [cases (%)]	27 (69.2)	7 (50.0)	20 (80.0)	0.075
Successful mobilization of CD34^+^ cells [cases (%)]	37 (94.5)	13 (92.9)	24 (96.0)	1.000

*rhG-CSF* recombinant human granulocyte colony-stimulating factor, *WBC* white blood cell. Other abbreviations as in Table 1

^a^The values are presented as median followed by range in parentheses; other values are presented as the number of patients followed by percentages in parentheses

### Toxicity

The most common toxicity was hematologic for both the CHOP and R-CHOP mobilization regimens: 20 (51.3%) patients experienced grade 4 neutropenia, 12 (30.8%) developed febrile neutropenia, and 3 (7.7%) had grade 3/4 thrombocytopenia. Anemia and vomiting were common but mild. Grade 3 anemia and vomiting occurred in 2 and 3 patients, respectively. PLT transfusions were administered to 3 patients, and a red blood cell (RBC) transfusion was only given to 1 patient. However, no fatal toxicities (grade ≥4) or transplantation-related mortality were observed in this study, and all the complications were mild and reversible within 24–48 h in all cases.

The neutrophil nadir from the first day of mobilization induced by the R-CHOP regimen occurred later than that induced by the CHOP regimen (*P* = 0.042), and the period from mobilization to the date of the PLT nadir was longer in the R-CHOP group than in the CHOP group (*P* = 0.008). However, the median neutrophil count and PLT count at the nadir were similar between the two groups. No significant differences in mobilization toxicities such as vomiting, diarrhea, and elevated alanine transaminase (ALT) were observed between the two groups. More details are outlined in Table 3.

**Table 3:** Toxicities after mobilization in 39 patients with high-risk non-Hodgkin’s lymphoma

Parameter	R-CHOP (*n* = 14)	CHOP (*n* = 25)	*P* value
Time from mobilization to the nadir of WBC (days)^a^	12 (9–14)	11 (0–16)	0.104
WBC count at nadir (×10^9^/L)^a^	1.24 (0.23–2.60)	1.30 (0.06–3.70)	0.856
Time from mobilization to the nadir of neutrophils (days)^a^	12.5 (9–15)	11 (5–15)	0.042
Neutrophil count at nadir (×10^9^/L)^a^	0.46 (0–1.60)	0.50 (0–2.41)	0.915
Time from mobilization to the nadir of PLT (days)^a^	14.5 (11–19)	13.0 (6–16)	0.008
PLT count at nadir (×10^9^/L)^a^	97.5 (51–171)	97.0 (12–193)	0.797
PLT transfusions [cases (%)]	1 (7.1)	2 (8.0)	0.748
Vomiting [cases (%)]			0.805
Grade 1/2	8 (57.1)	18 (72.0)	
Grade 3	1 (7.1)	2 (8.0)	
Febrile neutropenia [cases (%)]	3 (21.4)	9 (36.0)	0.477
Diarrhea [cases (%)]	0	4 (16.0)	0.387
ALT elevation [cases (%)]	5 (35.7)	15 (60.0)	0.234
Anemia [cases (%)]			0.212
Grade 1/2	7 (50.0)	20 (80.0)	
Grade 3	1 (7.1)	1 (4.0)	

*PLT* platelets, *ALT* alanine transaminase. Other abbreviations as in Tables 1 and 2

^a^The values are presented as median followed by range in parentheses; other values are presented as the number of patients followed by percentages in parentheses

### Conditioning regimens and engraftment

All 39 patients received transplantation, even the 2 patients who failed APBSC mobilization. Of the 39 patients, 28 (71.8%) received BEAC, 6 (15.4%) received BEAM, 3 (7.7%) received CBV, and 2 (5.1%) received CE-TBI as conditioning regimens.

The median time from APBSC infusion to reaching a WBC count of at least 1.5 × 10^9^/L for two consecutive days was 11 days (range 8–14 days), and the median time for PLT recovery was 12 days (range 7–32 days). The median duration of rhG-CSF support was 7 days (range 3–25 days). No differences were observed in the WBC and PLT recovery time between the two groups (Table 4).

**Table 4:** Post-transplantation engraftment and responses in 39 patients with high-risk non-Hodgkin’s lymphoma

Parameter	R-CHOP (*n* = 14)	CHOP (*n* = 25)	*P* value
Time from APBSC infusion to WBC engraftment (days)^a^	11 (9–13)	11 (8–14)	0.692
Time from APBSC infusion to PLT engraftment (days)^a^	12.5 (7–18)	12.0 (9–32)	0.988
Duration of rhG-CSF support from APBSC infusion to engraftment (days)^a^	8.5 (4–12)	7.0 (3–25)	0.410
CR for B-cell lymphoma [cases (%)]^b^	14 (100)	4 (50)	0.010

*APBSC* autologous peripheral blood stem cells, *rhG-CSF* recombinant human granulocyte colony-stimulating factor; CR, complete response. Other abbreviations as in Tables 1, 2 and 3

^a^The values are presented as median followed by range in parentheses; other values are presented as the number of patients followed by percentages in parentheses

^b^All the 14 patients in R-CHOP group and 8 patients in CHOP group had B-cell lymphoma

### Responses and survival

Of the 22 patients who failed to reach a CR before mobilization, 1 progressed and 8 maintained their PR status in the CHOP group, 13 converted to CR after mobilization, with 7 in the CHOP group and 6 in the R-CHOP group. The CR rate of patients with B-cell lymphoma was higher in the R-CHOP group than in the CHOP group (*P* = 0.010, Table 4). None of the 17 patients who achieved a CR before mobilization relapsed during mobilization.

**Fig. 1:**
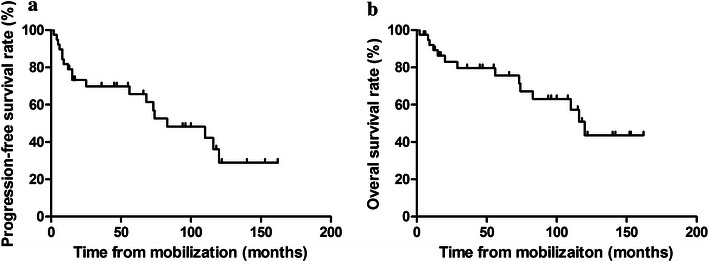
Survival curves of all patients with high-risk non-Hodgkin’s lymphoma mobilized by cyclophosphamide, doxorubicin, vincristine, and prednisolone (CHOP) alone or in combination with rituximab (R-CHOP). **a** Progression-free survival; **b** overall survival

The median follow-up time was 56 months (range 2–162 months); the 5-year PFS and OS rates for all patients were 65.7% (Fig. 1a) and 75.6% (Fig. 1b), respectively. No significant differences in OS or PFS were observed between the R-CHOP and CHOP groups.

### Factors associated with mobilization yield

**Fig. 2:**
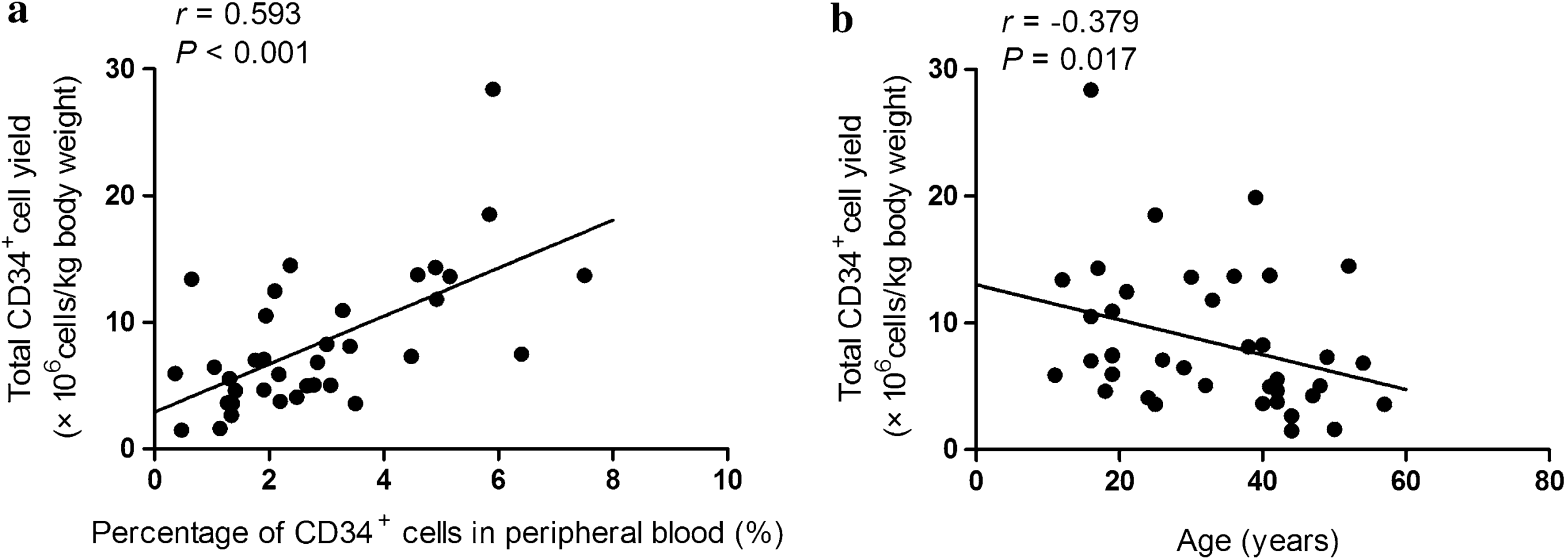
Factors associated with mobilization yield in patients with high-risk non-Hodgkin’s lymphoma. **a** Positive correlation between total CD34^+^ cell yield and the percentage of CD34^+^ cells in peripheral blood on the first day of apheresis. **b** Negative correlation between total CD34^+^ cell yield and age

In the univariate analysis, we observed a significant linear correlation between the percentage of CD34^+^ cells in peripheral blood on the first day of apheresis and the total CD34^+^ cell yield (Spearman *r* = 0.593, *P* < 0.001, Fig. 2a). Similarly, younger age (Spearman *r* = −0.379, *P* = 0.017, Fig. 2b) and male gender (*P* = 0.035) were associated with better CD34^+^ cell collection. Previous chemotherapy cycles (Spearman *r* = −0.337, *P* = 0.036), duration of rhG-CSF support for mobilization (Spearman *r* = −0.425, *P* = 0.008), and time from mobilization to apheresis (Spearman *r* = −0.453, *P* = 0.004) were negatively correlated with CD34^+^ cell collection. However, only age (*P* = 0.011) and the percentage of CD34^+^ cells in peripheral blood on the first day of apheresis (*P* < 0.001) had predictive values for the total CD34^+^ cell yield in multivariable analysis (Table 5).

**Table 5:** Multivariable analysis of the total CD34^+^ cell yield in 39 patients with high-risk non-Hodgkin’s lymphoma

Parameter	Total CD34^+^ cell yield (×10^6^ cells/kg body weight)		
	B	95% CI	*P* value
Percentage of CD34^+^ cells in peripheral blood on the first day of apheresis	1.886	1.104 to 2.669	<0.001
Previous chemotherapy cycles	−0.07	−1.386 to 1.245	0.914
Duration of rhG-CSF support	−0.829	−1.685 to 0.028	0.058
Time from mobilization to apheresis	−0.736	−1.544 to 0.073	0.073
Age	−3.718	−6.521 to −0.914	0.011

*CI* confidence interval. Other abbreviation as in Table 2

## Discussion

In the present study, the dose-adjusted CHOP and R-CHOP regimens were shown to be feasible and well tolerated in mobilizing sufficient APBSCs in NHL patients. Of this study population, 95% of patients were successful in achieving the target CD34^+^ cell harvest of ≥2 × 10^6^ cells/kg body weight, with a median CD34^+^ cell yield of 7.01 × 10^6^ cells/kg body weight (range 1.49–28.39 × 10^6^ cells/kg body weight). These results were superior to the published results for the traditional regimen of ICE combined with rituximab (88% success rate; median, 6.3 × 10^6^ cells/kg body weight; range 0–15.6 × 10^6^ cells/kg body weight) [23] or etoposide (94% success rate; median, 6.2 × 10^6^ cells/kg body weight; range 0–27.6 × 10^6^ cells/kg body weight) [24]. Possibly because of the excellent stem cell mobilization, all patients in our study successfully proceeded to transplantation. Although 51% of patients developed grade 4 neutropenia and 31% of patients experienced febrile neutropenia, no grade 4 toxicities or treatment-related deaths were observed, indicating the safety of this regimen. A PLT transfusion was given in 7% of patients, and an RBC transfusion was given in 2% of patients. These rates were significantly lower than those observed for patients mobilized by other regimens, such as etoposide (30 and 30%) [24] and ESHAP regimens (12 and 12%) [6].

The mobilization yield of CD34^+^ cells in the CHOP group appeared to be higher than that in the R-CHOP group, and this phenomenon was previously explained by the biological theory that perturbation of stromal-derived factor-1 during B-cell recovery after rituximab treatment retards neutrophil egress from the marrow [9]. However, the disadvantage of rituximab in terms of mobilization yield was offset by the high rate of successful mobilization. The efficacy of the R-CHOP regimen was similar to that of the CHOP regimen, with both resulting in a successful mobilization rate of over 90%. This finding was consistent with that in the study conducted by Lefrère et al. [8], who reported that 91 of 137 patients receiving rituximab exhibited a similar rate of successful mobilization as the non-rituximab group. When considering the safety and efficacy of ASCT, it is important to note that serious infections have been reported with rituximab administration because of delayed neutrophil and PLT recovery and late-onset neutropenia [10–12]. Nevertheless, Lefrère et al. [8] suggested that rituximab combined with chemotherapy was safe, without excess toxicity or delayed ASCT engraftment. In our study, the times to WBC and PLT recovery after transplantation were similar between the R-CHOP and CHOP groups, as were the toxicity and complications. Rituximab seemed to elevate the CR rate (*P* = 0.01) in B-cell lymphoma, which may be consistent with the results of previous studies [10, 13, 14]. Several other studies reported rituximab-induced interstitial lung disease, with some cases considered life-threatening [25, 26]. However, this toxicity was not observed in our study.

Our data showed that younger age and male gender were closely related with better mobilization yield, in agreement with the findings of Akhtar et al. [15]. Although PLT counts prior to mobilization have been significantly associated with the total CD34^+^ cell collection after mobilization in some studies [15, 18, 27], this association was not observed in our study, perhaps because of the smaller number of patients enrolled in our study. In addition, the higher percentage of CD34^+^ cells in peripheral blood on the first day of apheresis and the lower number of prior chemotherapy cycles resulted in a better mobilization yield in our study, consistent with the findings in several previous studies [1, 15, 18]. In light of the recent development of new classes of mobilization agents such as CXC chemokine receptor 4 (CXCR4) inhibitors and plerixafor, predictive factors are urgently needed to discern which patients are likely to benefit from the more efficient mobilization agents [28].

This study has several limitations. It was an uncontrolled, retrospective, single-center study, including selected patients treated over many years. However, we consider our results to be reliable for selecting an effective chemomobilization regimen because the clinical characteristics that might affect the APBSC mobilization yield were comparable with those in other studies, and apheresis procedures were performed according to similar guidelines as those in other studies.

## Conclusions

In conclusion, the present study showed that CHOP chemotherapy followed by rhG-CSF provides effective mobilization of APBSCs in patients with NHL and that the addition of rituximab to CHOP chemotherapy for APBSC mobilization is feasible. Prospective randomized trials are necessary to confirm the efficacy of this mobilization regimen.
